# Even the Smallest Non-Crop Habitat Islands Could Be Beneficial: Distribution of Carabid Beetles and Spiders in Agricultural Landscape

**DOI:** 10.1371/journal.pone.0123052

**Published:** 2015-04-10

**Authors:** Michal Knapp, Milan Řezáč

**Affiliations:** 1 Department of Ecology, Faculty of Environmental Sciences, Czech University of Life Sciences Prague, Prague, Czech Republic; 2 Group Functional Biodiversity, Crop Research Institute, Prague, Czech Republic; Roehampton University, UNITED KINGDOM

## Abstract

Carabid beetles and ground-dwelling spiders inhabiting agroecosystems are beneficial organisms with a potential to control pest species. Intensification of agricultural management and reduction of areas covered by non-crop vegetation during recent decades in some areas has led to many potentially serious environmental problems including a decline in the diversity and abundance of beneficial arthropods in agricultural landscapes. This study investigated carabid beetle and spider assemblages in non-crop habitat islands of various sizes (50 to 18,000 square metres) within one large field, as well as the arable land within the field, using pitfall traps in two consecutive sampling periods (spring to early summer and peak summer). The non-crop habitat islands situated inside arable land hosted many unique ground-dwelling arthropod species that were not present within the surrounding arable land. Even the smallest non-crop habitat islands with areas of tens of square metres were inhabited by assemblages substantially different from these inhabiting arable land and thus enhanced the biodiversity of agricultural landscapes. The non-crop habitat area substantially affected the activity density, recorded species richness and recorded species composition of carabid and ground-dwelling spider assemblages; however, the effects were weakened when species specialised to non-crop habitats species were analysed separately. Interestingly, recorded species richness of spiders increased with non-crop habitat area, whereas recorded species richness of carabid beetles exhibited an opposite trend. There was substantial temporal variation in the spatial distribution of ground-dwelling arthropods, and contrasting patterns were observed for particular taxa (carabid beetles and spiders). In general, local environmental conditions (i.e., non-crop habitat island tree cover, shrub cover, grass cover and litter depth) were better determinants of arthropod assemblages than non-crop habitat island size, indicating that the creation of quite small but diversified (e.g., differing in vegetation cover) non-crop habitat islands could be the most efficient tool for the maintenance and enhancement of diversity of ground-dwelling carabids and spiders in agricultural landscapes.

## Introduction

Carabid beetles and ground-dwelling spiders inhabiting agroecosystems are beneficial organisms with a potential for the biological control of pest species [1–4]. Intensification of agricultural management during recent decades in some areas has led to many potentially serious environmental problems including a decline in the diversity of beneficial arthropods within arable lands [3–7]. Many carabid and spider species have highly specific habitat requirements [3,4,8]. These requirements are not met in simplified and intensively cultivated contemporary agricultural landscapes, resulting in the decline of some habitat specialists and shifts in the functional traits of arthropod assemblages [9–11].

Interestingly, arthropod assemblages in agricultural landscapes appear to be affected by landscape structure at least as seriously as by local conditions (e.g., pesticide application or intensity of agricultural operations), and landscape structure substantially modifies the effects of local environmental conditions on arthropod assemblages [10,12–15]. In general, higher landscape heterogeneity is linked to enhanced species and functional diversity [12,14,16–18]. Thus, it is not surprising that the conservation of existing non-crop (semi-natural) habitats and the creation of new habitats appears to be one of the most efficient conservation practices applied in intensively managed agricultural landscapes [18–23].

Non-crop habitats within agricultural landscapes host unique species, i.e., species not present in cultivated areas [24]. Moreover, non-crop areas provide opportunities, e.g., overwintering location, food resource or sites for reproduction, even to species inhabiting arable land [25,26]. The distance to non-crop habitats substantially affects the species composition of predatory arthropod assemblages in cultivated areas because individuals from non-crop habitats disperse to neighbouring cultivated areas [27]. It is evident that non-crop habitat islands situated within (inside) cultivated areas are the most effective in providing assemblages from cultivated areas with “non-crop services” because these habitat islands have long boundaries shared with cultivated areas and should be situated in central parts of cultivated areas far away from their edges. On the other hand, assemblages from non-crop habitat islands isolated inside large cultivated areas are likely more seriously affected by the surrounding cultivated habitat (e.g., arthropod spillover from arable fields; [28]). Thus, it is not clear whether small non-crop habitat islands are able to provide “non-crop services” and to host arthropod assemblages different from assemblages inhabiting arable fields.

Patch size has received considerable attention in ecology and biodiversity conservation research (e.g., [29,30]). However, the majority of studies have addressed quite large habitat patches (e.g., ones to thousands of hectares; [31–33]), and studies focused on small patches (e.g., tens of square metres) are much rarer (e.g., [25]). Non-crop habitat islands within cultivated areas are disliked by farmers because the islands occupy the usable land for production purposes. Thus, the habitat island size is important from a farmer’s point of view, and small-sized non-crop habitat islands within cultivated areas are more acceptable to farmers than large-sized islands. Moreover, knowledge on relative importance of habitat island size and local habitat characteristics (e.g., vegetation cover) on ground-dwelling arthropods, which could be essential for efficient conservation management in agricultural landscapes, are limited (but see [32,34,35]).

Agroecosystems are extremely dynamic systems with high frequencies of large-scale disturbances (e.g., crop harvest, tillage, pesticide application) and large-scale changes in vegetation structure from year to year due to crop rotation [22,36]. The conditions experienced by organisms inhabiting agroecosystems, especially by those living within arable fields, are thus highly temporally variable within and between particular seasons. The spatial distribution of species is thus apparently unstable over time, which could complicate the application of conservation management actions aiming to improve the biological control of crop pests or to enhance biodiversity in agricultural landscapes [37,38]. Unfortunately, the large majority of studies investigating the effects of non-crop habitats on spatial distribution of arthropods in agroecosystems do not focus on temporal variation in species spatial distribution (e.g., [17,21,39,40]; but see [41]).

The aim of this study is to investigate the effects of the non-crop habitat island size (area) and structure on epigeal arthropods. In particular, we are interested in the effects of habitat type (arable field or non-crop habitat island) and non-crop habitat island size on the abundance, species richness and species composition of carabid beetles and ground-dwelling spiders. To distinguish between the effects of local environmental conditions and the effects of patch size, non-crop habitat island characteristics (tree cover, shrub cover, grass cover and litter depth) are included in the models, and a variance partitioning procedure is employed to assess the relative importance of particular factors. Separate analyses are performed for non-crop habitat specialists, i.e., species not occurring within arable land sites in this study, to verify or rule out the possibility that responses of ground-dwelling arthropod assemblages in non-crop habitat islands are driven by species spilling over from surrounding arable land. Moreover, the temporal variability in the observed patterns is investigated by analysing the effects of sampling period (spring and early summer vs. peak summer) and the interactions between sampling period and the other investigated independent variables.

## Materials and Methods

### Study site and data collection

**Fig 1:**
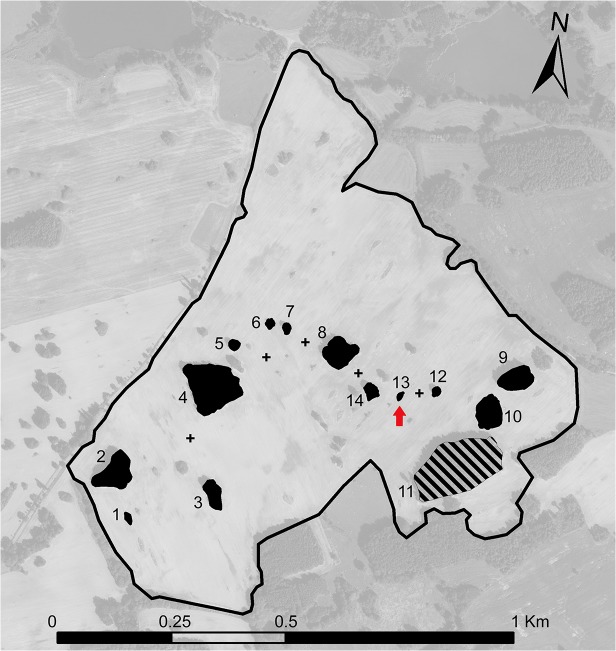
Map of the study site. Investigated non-crop habitat islands are highlighted in black. The red arrow indicates the smallest investigated island (with an area of 50 square metres). The hatched island was ultimately omitted from data analyses due to substantial trap destruction by large mammals. Crosses indicate the positions of arable field sites investigated in this study.

The study was performed within one large arable field (ca. 64 hectares) situated in the central part of the Czech Republic near Vysoký Chlumec (GPS: N 49°36’36.337”; E 14°24’59.94”). This field is unique due to the presence of a high number of non-crop habitat islands of various sizes within the field (Fig 1). The field is conventionally managed using following crop rotation scheme: winter wheat; maize; winter wheat; winter barley; winter rape. Organic fertilizers (cattle manure) are applied once each two years in dosage 30 tons per hectare. Mineral fertilizers are applied each year in dosage 0.5 ton per hectare (typically DAM 390, which contain 30% of nitrogen utilizable by plants). Pesticides are applied in dosage ca. 3 litres per hectare per year (mainly Roundup Rapid, Lentipur 500 FW, Stabilan 750 SL and Logran 20 WG). The soil texture is matching to silt loam and the soil has quite neutral acidity (pH = 6.0). The content of important chemical elements (in mg per kg of top soil) is following: phosphorus 144; potassium 250; magnesium 239; calcium 1873. The soil humidity is quite optimal for agricultural production (due to optimal soil texture and mean annual precipitation of 650 mm) and there are no extreme conditions, e.g., regular flooding. The non-crop habitat islands are vegetated mainly by trees (oaks, locusts and pines are dominating) and shrubs (e.g., wild rose, blackthorn), but the smaller islands are also partially covered by herbs (e.g., various grasses). Soil humidity within islands is a little bit lower than in arable land because more sandy soil and elevated ground level (islands are typically elevated by 20–50 cm).

To compare activity density, species richness and species composition of assemblages originating from various sized islands and arable sites, 14 non-crop habitat islands (with areas of 50 to 18,000 square metres) and five arable land sites (in between habitat islands; Fig 1) were selected and sampled for ground-dwelling arthropods using pitfall traps. No permits were required for the described study (field sampling of ground-dwelling arthropods). The study complied with all relevant national regulations. Landowner provided us with the permission to enter the area and collect beetles and spiders. Three pitfall traps were installed in the central part (centroid) of each non-crop habitat island (in a triangle array; traps were three metres from each other) and two pitfall traps per arable land site (traps were three metres from each other). We decided to employ lowered number of traps in arable land sites based on our previous experiences. Traps situated in arable land have lower probability of destruction by large mammals and arthropod assemblages inhabiting this habitat are more homogeneous, i.e. have low spatial species turnover (traps placed 3 metres from each other provide very similar samples). The pitfall traps were made of 0.5 l transparent plastic cups (round opening, 9.4 cm in diameter) filled with a 25% aqueous solution of propylene glycol as a preservative fluid (for details see [42]). The traps were covered by a non-transparent metal roof to protect the trap contents against precipitation. The field sampling was conducted in 2009 in two subsequent five-week sampling periods. The first sampling period started at the end of April, and the second sampling period started at the end of June. The rape grown on the field in 2009 was harvested immediately after the end of the second sampling period (early August). Thus, the first sampling period represents the stage of rape growing, and the second sampling period represents the stage of rape ripeness.

For each non-crop habitat island site, several environmental variables were measured. Non-crop habitat island area was estimated from aerial photographs using tools implemented in geographic information system (Esri ArcGIS 10.2.1 for Desktop). Tree cover (estimated percentage of island area covered by trees), shrub cover (estimated percentage of island area covered by shrubs), grass cover (estimated percentage of island area covered by grass) and litter depth (measured in cm; mean value was calculated based on ten measurements carried out in various parts of a particular island) were assessed for each island during the field survey.

### Arthropod sample processing

Samples from each pitfall trap were transferred to the 70% ethanol immediately after their collection, transported to the laboratory and stored in a freezer. Subsequently, the pitfall trap samples were sorted to higher taxa. Spiders (Araneae) and ground beetles (Coleoptera: Carabidae) were identified to species based on identification guides [43,44]. Juvenile specimens of spiders were excluded from the dataset because the majority of these juveniles were impossible to identify to the species level. The nomenclature of spiders follows that of Platnick [45]; the nomenclature of ground beetles follows that of Löbl and Smetana [46]. Voucher specimens of spiders were deposited in the private collection of Milan Řezáč (Prague) and those of carabid beetles were deposited in the private collection of Michal Knapp (Prague). It is important to note that pitfall traps are not able to sample all epigeal arthropod species (some are systematically missing in pitfall trap samples; Knapp et al. in prep.) and pitfall trap samples are generally biased towards large species [47]. Thus number of collected species is called “recorded species richness” (as we are aware that there are additional species not recorded by pitfall traps) and number of collected specimens is called “activity density” (as pitfall trap catches reflect movement activity as well as population density) throughout the following text.

### Data analyses

Samples originating from the largest island (no. 11 in Fig 1) were omitted from the analyses due to repeated trap destruction by large mammals. Prior to the statistical analyses, the data from three traps installed at a particular non-crop habitat island and two traps installed at a particular arable land site were pooled, resulting in 13 pooled island samples and 5 pooled arable land samples per sampling period. Data pooling was necessary to preclude pseudoreplication [48] and because the non-crop habitat island characteristics (tree cover, shrub cover, grass cover, litter depth and island area) were measured at the site level. All analyses were performed separately for carabid beetles and for spiders. All univariate analyses were performed in R 3.0.3 [49]. Multivariate analyses were performed in Canoco for Windows 5.0 software [50]. Species data for all multivariate analyses were logarithmically (log_10_(x+1)) transformed prior to the analyses.

#### Complete dataset including all collected species

To analyse the effects of non-crop habitat island characteristics and changes of their importance in time on activity density and recorded species richness, generalized linear models with a negative binomial of errors (GLM) were employed. Negative binomial distribution of errors is generally proper to count data (i.e. number of specimens or species per trap) and is especially suitable for models with overdispersion [48,51], which was the case in majority of models fitted in this study. To analyse the effects of non-crop habitat island characteristics and sampling timing on species composition, canonical correspondence analysis (CCA) was used. Tree cover, shrub cover, grass cover, litter depth, island area, sampling timing and interactions between sampling timing and particular non-crop habitat island characteristics were used as independent variables in the full models. A backward selection procedure based on deletion tests (Chi^2^ test) was employed to select for final GLMs. A forward selection procedure based on Monte-Carlo permutation tests (999 permutations applied) was used to select for final model in multivariate analyses (CCA). Significant terms (P < 0.05) were included in the final models. Variance partitioning was applied to the final models to highlight the importance of particular significant terms. To simplify the variance partitioning results, variance partitioning by groups of variables was applied: 1^st^ group = sampling period; 2^nd^ group = local environmental conditions (i.e., tree cover, shrub cover, grass cover, litter depth); 3^rd^ group = non-crop habitat island area.

To analyse the differences in activity density (number of specimens collected) and recorded species richness between non-crop habitat islands and arable land sites and between sampling periods, generalized linear models with a negative binomial distribution of errors were used. For purpose of these analyses activity density and recorded species richness of particular site were represented by mean number of specimens/species per trap to enable comparison between sites with various number of pitfall traps employed (3 traps in non-crop habitat islands and 2 traps in arable land sites). To analyse the effects of habitat type and sampling timing on species composition, a multivariate technique (CCA) was employed. CCA was based on pooled samples as “standardization by samples” is inherent for this analysis (i.e. relative instead of absolute abundances are analysed; [50]). Habitat type (non-crop habitat island or arable land site), sampling timing (first or second sampling period) and interaction between habitat type and sampling timing were used as independent variables in the models.

To visualise the differences in species composition between arable land sites and non-crop habitat islands of various sizes, detrended correspondence analysis (DCA) based on pooled data was employed. Individual non-crop habitat island sites were classified according to their area (large circles in ordination diagram = large islands), and temporal shifts in species composition of particular sampling sites are shown.

#### Partial dataset including solely non-crop habitat specialists

To verify/rule out the possibility that response of non-crop habitat island assemblages to island area and habitat characteristics is driven by species spilling over from arable land, we decided to analyse separately the subset of species that were absent in arable land sites in this study (see S1 and S2 Tables). Such species were called “non-crop habitat specialists”. To analyse the effects of non-crop habitat island characteristics (tree cover, shrub cover, grass cover, litter depth), non-crop habitat island area and sampling period on the activity density and recorded species richness of non-crop habitat specialists, generalized linear models with a negative binomial distribution of errors were employed. To analyse the effects of non-crop habitat island characteristics and sampling timing on species composition of non-crop habitat specialist carabids and spiders, CCA was applied. Term selection procedures and variance partitioning were applied in the same way as in the case of analysing the complete dataset (see above).

## Results

In total, 6,772 carabid beetles of 66 species and 3,081 adult spiders of 90 species were collected. Seventeen carabid species were collected solely in non-crop habitat islands and 8 species were unique to arable land sites (for details see S1 Table). Fifty-nine spider species were collected solely in non-crop habitat islands and 9 species were unique to arable land (for details see S2 Table).

### Total activity density

**Fig 2:**
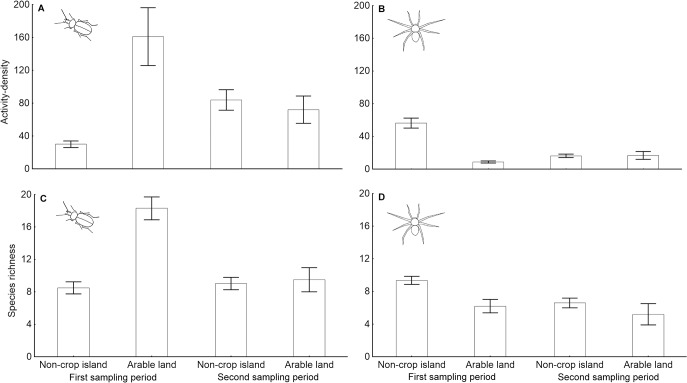
The effects of habitat type and sampling period on the activity density and recorded species richness of ground-dwelling arthropods. Mean number of A) carabid beetles and B) spiders collected per trap (i.e., activity density) and mean recorded species richness of C) carabid beetles and D) spiders per trap ± SE are shown for both sampling periods investigated (spring to early summer and peak summer).

**Fig 3:**
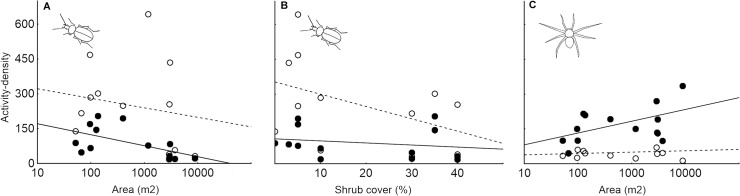
The effects of non-crop habitat island characteristics and sampling period on the activity density of ground-dwelling arthropods. The relationships between A) number of collected carabids and non-crop habitat island area, B) number of collected carabids and non-crop habitat island shrub cover and C) number of collected spiders and non-crop habitat island area are shown. Full circles (solid line) represent the first sampling period (spring to early summer) and open circles represent the second sampling period (peak summer).

The number of carabids collected (their activity density) was significantly affected by habitat type (Chi^2^_1_ = 8.71; P = 0.003), sampling period (Chi^2^_1_ = 4.96; P = 0.026) and the interaction between habitat type and sampling period (Chi^2^_1_ = 12.38; P < 0.001). In the first sampling period (spring to early summer), carabid beetles were more numerous in arable field sites compared to non-crop habitat islands, whereas the difference between habitat types disappeared in the second sampling period (summer; see Fig 2A). The carabid activity density within non-crop habitat islands significantly increased in the second sampling period (Chi^2^_1_ = 15.08; P < 0.001), decreased with increasing non-crop habitat island size (Chi^2^_1_ = 5.95; P = 0.015; Fig 3A) and was negatively affected by shrub cover (Chi^2^_1_ = 3.89; P = 0.048; Fig 3B). The most influential variable affecting the carabid activity density was sampling period; its net effects explained 31.5% of the total variation in the data (for details see S1 Fig).

The activity density of adult spiders was significantly affected by the sampling period (Chi^2^_1_ = 34.22; P < 0.001), habitat type (Chi^2^_1_ = 13.96; P < 0.001) and the interaction between habitat type and sampling period (Chi^2^_1_ = 23.05; P < 0.001). In contrast to carabids, spiders were more frequently caught in non-crop habitat islands than in arable field sites in the first sampling period, whereas the difference between habitat types disappeared in the second sampling period in a similar way as in carabids (Fig 2B). The activity density of spiders within non-crop habitat islands significantly decreased in the second sampling period (Chi^2^_1_ = 42.99; P < 0.001; the variation explained by its net effects was 57.3%; for details see S2 Fig) and was positively related to non-crop habitat island size (Chi^2^_1_ = 5.04; P = 0.025; Fig 3C).

### Total recorded species richness

**Fig 4:**
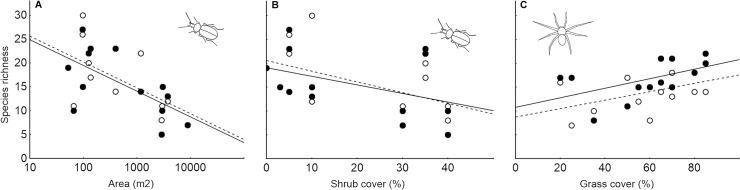
The effects of non-crop habitat island characteristics and sampling period on the recorded species richness of ground-dwelling arthropods. The relationships between A) number of carabid species recorded and non-crop habitat island area, B) number of carabid species recorded and non-crop habitat island shrub cover and C) number of spider species recorded and non-crop habitat island grass cover are shown. Full circles (solid line) represent the first sampling period (spring to early summer) and open circles represent the second sampling period (peak summer).

Recorded carabid species richness was significantly affected by habitat type (Chi^2^_1_ = 12.44; P < 0.001) and the interaction between habitat type and sampling period (Chi^2^_1_ = 7.35; P = 0.007). The mean number of carabid species collected per trap in arable field sites was much higher than in non-crop habitat islands in the first sampling period. The difference in carabid species richness between habitat types also continued in the second sampling period but to a lesser extent (Fig 2C). The species richness of carabids inhabiting non-crop habitat islands was negatively affected by non-crop habitat island size (Chi^2^_1_ = 21.79; P < 0.001, Fig 4A) and shrub cover (Chi^2^_1_ = 7.25; P = 0.007; Fig 4B). Based on variance partitioning, the most influential variable was non-crop habitat island area (its net effects explained 34.8% of the total variation in carabid species richness data; for details see S3 Fig).

The observed species richness of adult spiders was significantly affected by sampling period (Chi^2^_1_ = 6.38; P = 0.012) and habitat type (Chi^2^_1_ = 5.36; P = 0.021). The higher mean species richness of spiders was recorded in the first sampling period compared to the second sampling period, and more spider species were collected within non-crop habitat islands compared to arable land sites during both sampling periods (Fig 2D). The observed species richness of adult spiders inhabiting non-crop habitat islands was positively related to the non-crop habitat island area covered by grasses (Chi^2^_1_ = 6.51; P = 0.011; Fig 4C). Grass cover explained 21.1% of the total variation in spider species richness data.

### Total species composition

**Fig 5:**
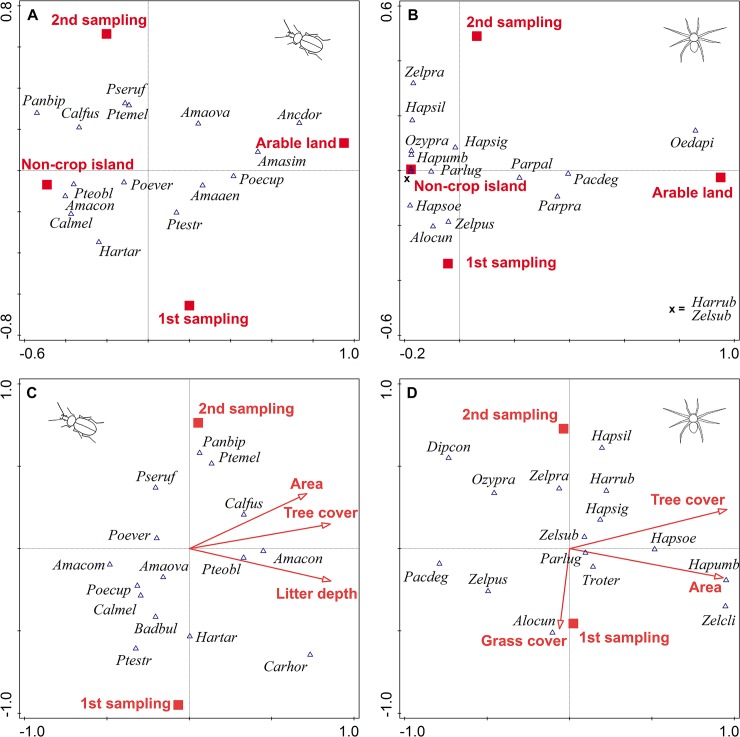
The effects of habitat type and non-crop habitat island characteristics on the species composition of ground-dwelling arthropod assemblages. The ordination diagrams based on the correspondence canonical analysis visualising A) the effects of habitat type and sampling period on carabid beetle assemblages, B) the effects of habitat type and sampling period on spider assemblages, C) the effects of non-crop habitat island characteristics and sampling period on carabid beetle assemblages and D) the effects of non-crop habitat island characteristics and sampling period on spider assemblages. Fifteen of the most influential species for each analysis are displayed and named using abbreviations made from the first three letters of the genus name followed by the first three letters of the species name (for full names see S1 and S2 Tables).

The species composition of both carabid and spider assemblages collected in this study was significantly affected by habitat type (carabids: pseudo-F = 6.6, P = 0.001; spiders: pseudo-F = 7.5, P = 0.001) and sampling period (carabids: pseudo-F = 2.8, P = 0.001; spiders: pseudo-F = 2.0, P = 0.001), but the interaction between habitat type and sampling period was not significant. The associations of selected species to non-crop habitat islands or arable sites and species prevalence in each sampling period are shown in Fig 5 (panels A and B).

The species composition of carabid assemblages within non-crop habitat islands was significantly affected by litter quantity (pseudo-F = 2.6, P = 0.001), tree cover (pseudo-F = 2.0, P = 0.001), non-crop habitat island area (pseudo-F = 2.3, P = 0.001) and sampling period (pseudo-F = 2.5, P = 0.001; Fig 5C). Based on the variance partitioning procedure, the non-crop habitat island characteristics (litter quantity and tree cover; their net effects together accounted for 7.0% of the total variation in species data) were the most important determinants of carabid assemblage species composition together with sampling period (its net effects explained 6.8% of the variation in species data; for details see S4 Fig).

The composition of spider assemblages inhabiting non-crop habitat islands was significantly affected by tree cover (pseudo-F = 3.6, P = 0.001), grass cover (pseudo-F = 1.6, P = 0.008), non-crop habitat island area (pseudo-F = 3.4, P = 0.001) and sampling period (pseudo-F = 1.7, P = 0.018; Fig 5D). The variance partitioning procedure revealed that the species composition of spider assemblages was mostly affected by non-crop habitat island characteristics (tree and grass cover; their net effects together accounted for 6.6% of the total variation in data; for details see S5 Fig).

**Fig 6:**
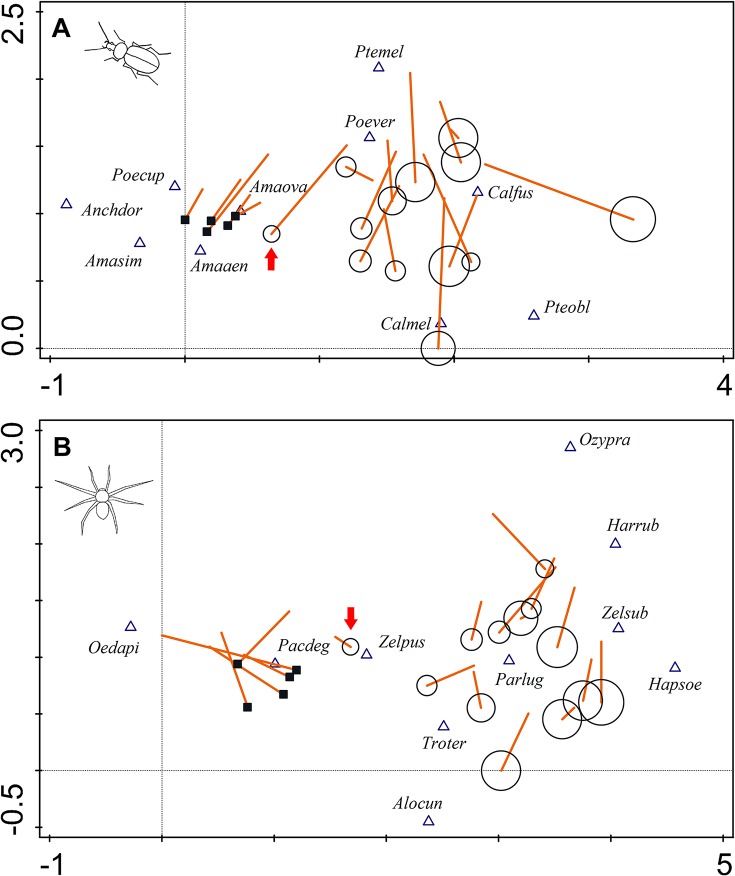
The temporal shift in the species composition of ground-dwelling arthropod assemblages inhabiting agricultural landscapes. The ordination diagrams based on the detrended canonical analysis visualising similarity in the species composition of A) carabid beetle and B) spider assemblages originating from particular sampling sites are shown. The position of a particular site sampled in the first sampling period (spring to early summer) is in the ordination space indicated by circle (non-crop habitat island) or square (arable field site) symbols. The temporal shift in species composition of a particular sampling site between the first and the second sampling period (peak summer) is indicated by the line originating in the centre of particular symbol. The size of circle symbols indicates the area of particular non-crop habitat islands. The red arrow indicates the smallest non-crop habitat island. The first two ordination axes explain 27.2% of the total variation in species data in the case of carabid beetles and 27.7% in the case of spiders. The ten most influential species are displayed and named using abbreviations made from the first three letters of the genus name followed by the first three letters of the species name (for full names see S1 and S2 Tables).

Unconstrained multivariate analyses (DCA) indicated that even the smallest non-crop habitat islands, with an area of tens of square metres, host carabid and spider assemblages more similar to assemblages from large non-crop habitat islands than to arable sites (with the exception of island no. 13, which is indicated by the arrow in Fig 6). Nonetheless, there were apparent some edge effects and species spillover from surrounding arable land in the smallest non-crop habitat islands as their central parts also hosted species prevalent in arable land, e.g., *Amara similata*, *Poecilus cupreus* and *Oedothorax apicatus*. By contrast, the large non-crop habitat islands were inhabited by several species, mainly forest specialists, which did not occur within the smallest islands, e.g., *Carabus hortensis*, *Harpalus quadripunctatus* and *Haplodrassus umbratilis*. Interestingly, the carabid assemblages originating from non-crop habitat islands became more similar to those from arable sites as the season progressed (from spring to summer; see lines indicating temporal shift in species composition of carabid assemblages in Fig 6A). In contrast to carabids, the species composition of spider assemblages originating from arable sites diverged in time from those originating from non-crop habitat islands (Fig 6B).

### Activity density, recorded species richness and species composition of non-crop habitat island specialists

**Fig 7:**
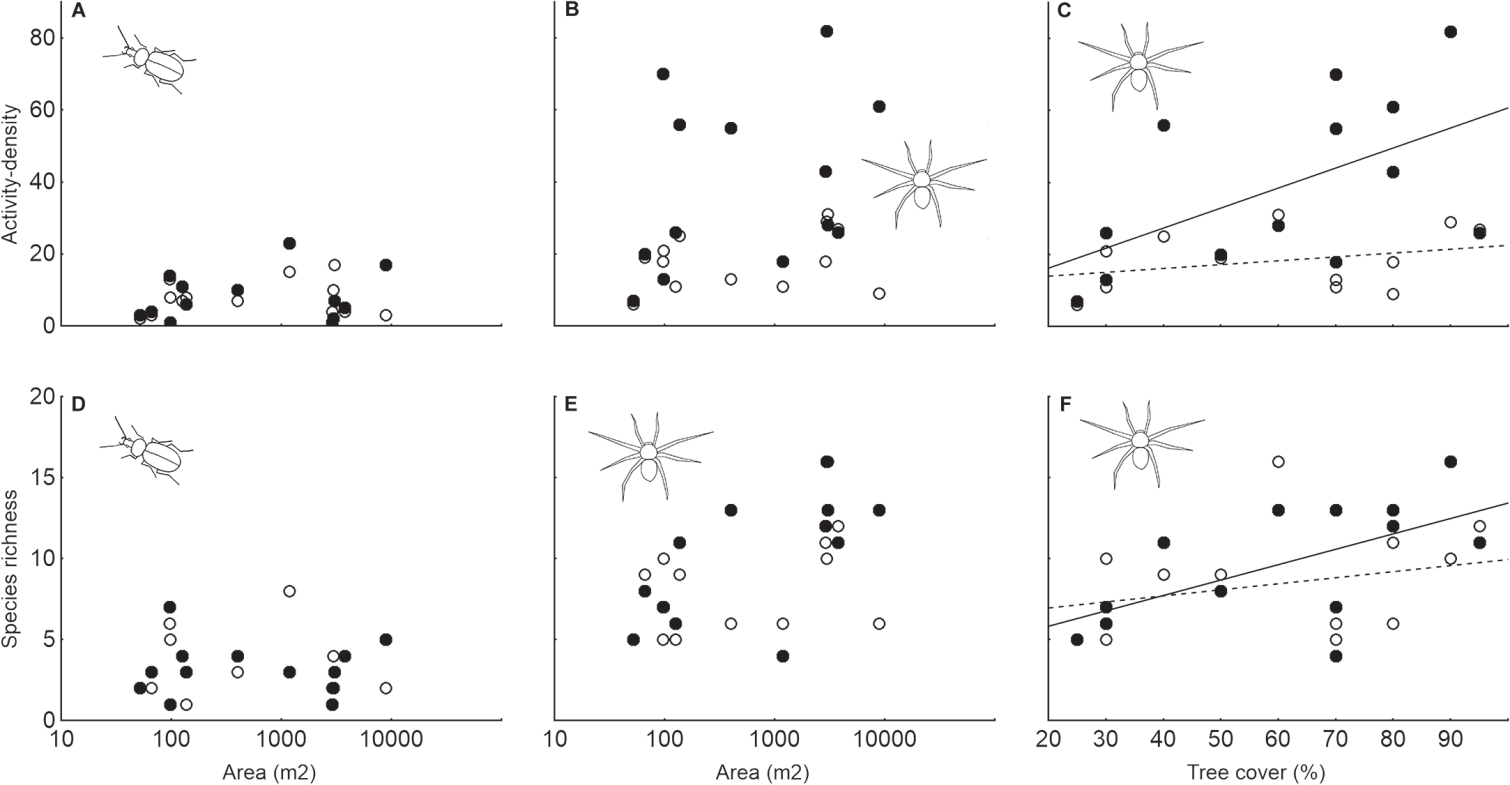
The effects of non-crop habitat island characteristics on the activity density and recorded species richness of non-crop habitat arthropod specialists. The relationships between A) activity density of carabid non-crop specialists and non-crop habitat island area, B) activity density of spider non-crop specialists and habitat island area, C) activity density of spider non-crop specialists and habitat island tree cover, D) number of carabid non-crop specialist species recorded and non-crop habitat island area, E) number of spider non-crop specialist species recorded and non-crop habitat island area, F) number of spider non-crop specialist species recorded and habitat island tree cover are shown. Full circles (solid line) represent the first sampling period (spring to early summer) and open circles represent the second sampling period (peak summer).

Carabid species not occurring within arable land had higher activity density in non-crop habitat islands with deeper litter layer (Chi^2^_1_ = 7.20; P = 0.007; S6 Fig). None of the other investigated variables had any significant effect on the activity density of specialists (including non-crop habitat island area; Fig 7A). There were no significant effects of the investigated variables on the species richness of non-crop habitat specialist carabids (including non-crop habitat island area; Fig 7D).

The activity density of non-crop specialist spiders was significantly affected by sampling period (Chi^2^_1_ = 17.45; P < 0.001) and was positively related to non-crop habitat tree cover (Chi^2^_1_ = 7.91; P = 0.005; Fig 7C) and non-crop habitat grass cover (Chi^2^_1_ = 4.44; P = 0.035; S7 Fig). The species richness of specialist spiders was positively related to non-crop habitat tree cover (Chi^2^_1_ = 6.62; P = 0.010; Fig 7F) and non-crop habitat grass cover (Chi^2^_1_ = 5.80; P = 0.016; S8 Fig). The non-crop habitat island areas were not included in the final model for non-crop specialist spider activity density and species richness (but see quite high correlation between non-crop habitat island area and tree cover of particular non-crop island; S11 Fig).

The species composition of carabid non-crop habitat specialists was significantly affected by litter quantity (pseudo-F = 2.6, P = 0.008), shrub cover (pseudo-F = 2.0, P = 0.014) and sampling period (pseudo-F = 1.8, P = 0.028; S9 Fig). The variance partitioning procedure revealed that the non-crop habitat island characteristics (litter quantity and shrub cover; their net effects together accounted for 9.7% of the total variation in species data) were the most important determinants of carabid non-crop habitat specialist composition. The species composition of spider non-crop habitat specialists was significantly affected by tree cover (pseudo-F = 2.8, P = 0.001), shrub cover (pseudo-F = 1.6, P = 0.020), grass cover (pseudo-F = 1.6, P = 0.024), non-crop habitat island area (pseudo-F = 2.6, P = 0.001) and sampling period (pseudo-F = 1.5, P = 0.038; S10 Fig). The non-crop habitat island characteristics (non-crop habitat island tree cover, shrub cover and grass cover; their net effects together accounted for 8.7% of the total variation in species data) were the most important determinants of spider non-crop habitat specialist composition.

## Discussion

Although based in just one field, results showed that in this system the non-crop habitat islands represented important landscape structures that substantially shape species distribution in agricultural landscapes. Even the smallest islands with areas of tens of square metres differed from arable land in the abundance (activity density), species richness and species composition of ground-dwelling arthropod assemblages. Non-crop habitat islands host many unique species that are not present within surrounding arable land.

Non-crop habitats likely contribute to pest suppression due to the beneficial arthropods within arable fields in their neighbourhood [52]. The carabid activity density observed in this study was substantially higher within the arable fields compared to the non-crop habitat islands in spring and early summer but became almost equal in peak summer. In contrast to carabids, the activity density of spiders was much higher in the non-crop habitat than within the arable land in the early season but also equalised in peak summer. These patterns indicate that there is substantial intra-annual variation in arthropod spatial distribution. Such variation could be driven by substantial changes in microclimate and food sources experienced by predatory arthropods within arable fields during the course of a season, which result from crop growth and subsequent harvest. Most carabid and spider species are sensitive to such changes in environmental conditions [3,4]. Moreover, changes in environmental conditions within matrix habitat can result in changes of edge responses of species within neighbouring non-crop habitats [53]. Species-specific ontogenetic development could also be particularly responsible for the temporal variation in the activity density of adult arthropods, as some species overwinter as adults that are then active from early spring, whereas other species overwinter as larvae and their adults emerge during the summer [4]. However, to distinguish between the effects of ontogeny and the effects of ongoing changes in environmental conditions, detailed analyses of the relationships between species ontogeny, their habitat preferences and temporal variation in their habitat use will be necessary. This may represent an interesting topic for future studies. It is important to note that difference in observed activity density between habitat types could not represent just difference in population density but could be also partially linked to habitat specific difference in species activity. It is well known that arthropod activity is tightly related to ambient temperature and thus activity is generally higher in open habitats with sparse vegetation especially during sunny days [23,54]. The contrasting patterns of carabid and spider activity density could also be caused by the direct interactions between these two groups of predators, as intra-guild predation between carabids and spiders is well documented from arable lands [1,55].

Carabid species richness was lower within the non-crop habitat islands compared to the arable land sites, and the opposite pattern was observed for spiders. However, from the biodiversity conservation point of view, the most important finding is that the non-crop habitat islands hosted a high number of unique spider as well as carabid species, which did not occur within the arable land. Such distinctness in species composition of assemblages originating from non-crop habitats and from arable land confirms the importance of non-crop habitat presence for biodiversity maintenance in landscapes with intensive agriculture [16,56,57].

The main aim of this study was to assess the importance of non-crop habitat island size in shaping ground-dwelling arthropod assemblages inhabiting these islands. The non-crop habitat island area significantly affected all the investigated aspects of carabid and spider total recorded assemblages, with the exception of total recorded spider species richness. On the other hand, the local environmental conditions (tree cover, shrub cover, grass cover, litter depth) were in general more influential than island area per se. Interestingly, when non-crop habitat specialists were analysed separately, there were no significant effects of non-crop habitat island area on any investigated assemblage parameter with the exception of the significant effect on specialist spider species composition. This finding contradicts our expectations and the general opinion that habitat specialists are more seriously (and positively) affected by habitat patch size than habitat generalists [29,31,58]. The negative effects of non-crop habitat area on total carabid species richness and activity density and the positive effects of non-crop habitat area on the total activity density of spiders appear to be driven mainly by generalist species inhabiting both arable land and non-crop habitat islands. Generalist carabid beetles, which were more numerous and species rich in arable land, probably spill over into non-crop habitat islands and resulting enhancement of activity density and species richness observed in central part of non-crop habitat islands is more pronounced in small islands with traps placed just several metres from adjacent arable land. It is important to note that a previous study on carabid beetles identified positive species-area relationship for core species (forest interior specialists) but the opposite for edge species [58]. Potential explanation of our unexpected results is also that “edge species” are probably classified in this study also as “non-crop habitat island specialists”. This is reasonable from the biodiversity conservation point of view as the presence of these edge species in agricultural landscapes is inherently linked to the presence of non-crop habitats.

It is also important to note that species richness recorded using pitfall traps represents species density (number of trappable species within limited area surrounding pitfall traps) and not absolute species richness (number of species inhabiting particular non-crop habitat island). Thus it is possible that carabids within our study system do not really avoid positive species-area relationship, but samples from large non-crop habitat islands represents solely species occurring within central parts of particular island, whereas edge and generalist species are almost completely missing from these samples. This pattern could be especially obvious in our study system where matrix (arable field) is highly productive, i.e. hosting many carabid species with high population densities, and non-crop habitat islands represent less productive habitats than surrounding matrix. On the other hand missing species-area relationship has been previously reported for spiders [34] and even negative species-area relationship has been previously reported for carabids [59].

The assemblages originating from small non-crop habitat islands were more similar to assemblages from large non-crop habitat islands than to those from arable land sites. These results indicate that even very small non-crop habitat islands situated inside arable fields are able to host unique assemblages and contribute to the enhancement of biodiversity at the local scale. The smallest non-crop habitat islands are most likely so small that edge effects affect assemblages inhabiting central parts of these islands [28,60]. On the other hand, edge effects around boundary between non-crop habitat and arable field appear to be quite short-range, especially towards the non-crop habitat interior [61], which ensures that even the islands covering tens of square metres are able to host assemblages different from these inhabiting arable land.

It stands to reason that farmers are not prone to sacrifice large areas of arable land for creation of non-crop habitat islands. However, many studies acknowledge the importance of non-crop habitats for the preservation of beneficial arthropod biodiversity in agricultural landscapes (e.g., [12,17,56]) and it has recently been shown by Rusch et al. [62] that the positive effects of the presence of non-crop habitats on pest control services cannot be substituted by crop rotation management. Fortunately, even small non-crop habitat patches within agricultural landscapes are able to support the biodiversity of various functional groups of organisms, e.g., plants, insect pollinators and predatory arthropods [19,25,63,64] and to offer the potential improvement of ecosystem services, e.g., pest suppression and crop pollination [25,52]. Spatial configuration of non-crop habitats within the agricultural landscape will be crucial for the functioning of these patches as resources of natural enemies and refuges for species avoiding arable land. Beneficial arthropods within arable land seem to profit from the presence of non-crop habitats only within a limited distance from their boundaries [65,66], indicating that the location of non-crop habitats within arable fields will ensure the highest effects on assemblages in surrounding arable land. On the other hand, the increasing distance of such non-crop habitat islands from nearest non-crop habitats will lead to lowered island colonisation due to increased spatial isolation and to potentially higher negative effects linked to arthropod local extinction and spillover from surrounding arable land [28,67,68]. Unfortunately, current knowledge on the movement of beneficial arthropods between various habitat types and on arthropod spatial distribution within agricultural landscapes in general are limited [27]. Thus, future research focused on the effects of non-crop habitat island spatial position in combination with its size and composition of surrounding landscape is needed to enable optimisation of spatial distribution of newly created non-crop habitat islands.

The significant effect of non-crop habitat island characteristics other than island area (i.e., tree cover, shrub cover, grass cover and litter depth) in shaping ground-dwelling arthropod assemblages and the high portion of variance explained by these variables stress that non-crop habitat patches of similar size, but that differ in vegetation structure, could host substantially different arthropod assemblages. Jonsson et al. [32] showed that invertebrate diversity could be linked to vegetation diversity and does not have to follow positive species-area relationship on real islands. The complementarity of various non-crop habitat types in forming the total species richness of temperate agricultural landscapes was recently shown by Diekoetter and Crist [56]. Therefore, newly created non-crop habitat islands should not be uniform, and the existing non-crop habitats should be managed in a way that leads to diversification of their properties. Unfortunately, some habitat characteristics are unavailable for small islands, e.g., real forest interior. Thus, properly managed small non-crop habitat islands are able to substantially enhance biodiversity of agricultural landscape, but some larger non-crop habitats are necessary to maximise arthropod diversity.

It will be highly valuable to replicate similar studies investigating effects of non-crop habitat island size and local environmental characteristics on ground-dwelling arthropod assemblages in study systems with different climatic and local environmental conditions (matrix and non-crop habitat island properties, e.g., soil fertility and structure). Such replications will enable to generalize the results of this study or to identify specific patterns linked to particular environmental conditions.

## Conclusions

Non-crop habitat islands situated inside arable land host many unique ground-dwelling arthropod species that are not present within surrounding arable land. Even the smallest non-crop habitat islands with areas of tens of square metres were inhabited by assemblages different from those inhabiting arable land sites and thus enhance the biodiversity at local scale. The non-crop habitat area substantially affected the majority of the investigated characteristics of recorded ground-dwelling arthropod assemblages; however, the effects were weakened when non-crop habitat specialists (species collected solely within non-crop habitat islands) were analysed separately. The local environmental conditions (i.e., non-crop habitat island tree cover, shrub cover, grass cover and litter depth) were better determinants of carabid and spider assemblages than was non-crop habitat island size. The creation of quite small but diversified (e.g., differing in vegetation cover) non-crop habitat islands thus seems to be potentially efficient tool for the maintenance or even enhancement of carabid and spider diversity within arable fields. Interestingly, there were some contrasting patterns in the activity density of carabid beetles and spiders (spiders were more frequently collected in non-crop habitats, whereas carabids were more frequently collected in arable land; spider activity density increased with non-crop habitat island area, whereas the activity of carabid beetles decreased). This could be a result of intra-guild predation; nonetheless, further detailed studies would be a worthwhile effort.

## Supporting Information

S1 Dataset Raw species and environmental data in xlsx format. (XLSX)

S1 Table Complete list of recorded carabid species classified by their habitat preferences. Species highlighted in green were sampled solely within arable land sites (= arable land specialists), species highlighted in red were sampled solely within non-crop habitat islands (= non-crop habitat specialists) and species written in black were sampled in both habitat types (= generalist species). Abbreviations are shown for species displayed in ordination diagrams.
(PDF)

S2 Table Complete list of recorded spider species classified by their habitat preferences. Species highlighted in green were sampled solely within arable land sites (= arable land specialists), species highlighted in red were sampled solely within non-crop habitat islands (= non-crop habitat specialists) and species written in black were sampled in both habitat types (= generalist species). Abbreviations are shown for species displayed in ordination diagrams.
(PDF)

S1 Fig Relative importance of particular variables affecting the total activity density of carabid beetles within non-crop habitat islands. Variance partitioning was based on the following groups of variables: 1^st^ group = sampling period; 2^nd^ group = local environmental conditions (i.e., tree cover, shrub cover, grass cover, litter depth); 3^rd^ group = non-crop habitat island area. A particular group of variables is omitted when it includes no significant variable. Net effects and shared variation (percentage of total variance) explained by particular groups of variables are shown.
(DOCX)

S2 Fig Relative importance of particular variables affecting the total activity density of adult spiders within non-crop habitat islands. Variance partitioning was based on the following groups of variables: 1^st^ group = sampling period; 2^nd^ group = local environmental conditions (i.e., tree cover, shrub cover, grass cover, litter depth); 3^rd^ group = non-crop habitat island area. A particular group of variables is omitted when it includes no significant variable. Net effects and shared variation (percentage of total variance) explained by particular groups of variables are shown.
(DOCX)

S3 Fig Relative importance of particular variables affecting the total recorded species richness of carabid beetles within non-crop habitat islands. Variance partitioning was based on the following groups of variables: 1^st^ group = sampling period; 2^nd^ group = local environmental conditions (i.e., tree cover, shrub cover, grass cover, litter depth); 3^rd^ group = non-crop habitat island area. A particular group of variables is omitted when it includes no significant variable. Net effects and shared variation (percentage of total variance) explained by particular groups of variables are shown.
(DOCX)

S4 Fig Relative importance of particular variables affecting the species composition of carabid beetle assemblages recorded within non-crop habitat islands. Variance partitioning was based on the following groups of variables: 1^st^ group = sampling period; 2^nd^ group = local environmental conditions (i.e., tree cover, shrub cover, grass cover, litter depth); 3^rd^ group = non-crop habitat island area. Net effects and shared variation (percentage of total variance) explained by particular groups of variables are shown.
(DOCX)

S5 Fig Relative importance of particular variables affecting the species composition of spider assemblages recorded within non-crop habitat islands. Variance partitioning was based on the following groups of variables: 1^st^ group = sampling period; 2^nd^ group = local environmental conditions (i.e., tree cover, shrub cover, grass cover, litter depth); 3^rd^ group = non-crop habitat island area. Net effects and shared variation (percentage of total variance) explained by particular groups of variables are shown.
(DOCX)

S6 Fig The relationship between the activity density of non-crop habitat specialist carabids and non-crop habitat island litter depth. Full circles (solid line) represent the first sampling period (spring to early summer) and open circles represent the second sampling period (peak summer).
(DOCX)

S7 Fig The relationship between the activity density of non-crop habitat specialist spider adults and non-crop habitat island grass cover. Full circles (solid line) represent the first sampling period (spring to early summer) and open circles represent the second sampling period (peak summer).
(DOCX)

S8 Fig The relationship between the recorded species richness of non-crop habitat specialist spider adults and non-crop habitat island grass cover. Full circles (solid line) represent the first sampling period (spring to early summer) and open circles represent the second sampling period (peak summer).
(DOCX)

S9 Fig The effects of sampling period, shrub cover and litter depth on the species composition of non-crop habitat specialist carabid assemblages recorded within non-crop habitat islands. The ten most influential species for the analysis (CCA) are displayed and named using abbreviations made from the first three letters of the genus name followed by the first three letters of the species name (for full names see S1 Table).
(DOCX)

S10 Fig The effects of sampling period, shrub cover, grass cover, tree cover and habitat island area on the species composition of non-crop habitat specialist spider assemblages recorded within non-crop habitat islands. The ten most influential species for the analysis (CCA) are displayed and named using abbreviations made from the first three letters of the genus name followed by the first three letters of the species name (for full names see S2 Table).
(DOCX)

S11 Fig The correlation matrix of non-crop habitat island characteristics investigated in this study. The relationships between tree cover (treecov), grass cover (grasscov), shrub cover (shrubcov), litter depth (litter) and non-crop habitat island area (logarea) are displayed. The matrix was generated using “pairs.panels” function from the “psych” package for R.
(DOCX)
