# Supplementary material for: Even the Smallest Non-Crop Habitat Islands Could Be Beneficial: Distribution of Carabid Beetles and Spiders in Agricultural Landscape
Source: PLoS One. 2015 Apr 10;10(4):e0123052. doi: 10.1371/journal.pone.0123052 (PMC4393288; doi:10.1371/journal.pone.0123052)
Supplement: S1 Table — Species highlighted in green were sampled solely within arable land sites (= arable land specialists), species highlighted in red were sampled solely within non-crop habitat islands (= non-crop habitat specialists) and species written in black were sampled in both habitat types (= generalist species). Abbreviations are shown for species displayed in ordination diagrams. (PDF) [file pone.0123052.s002.pdf]

S1 Table

Complete list of recorded carabid species classified by their habitat preferences. Species highlighted in green were sampled solely within arable land sites (= arable land specialists), species highlighted in red were sampled solely within non-crop habitat islands (= non-crop habitat specialists) and species written in black were sampled in both habitat types (= generalist species). Abbreviations are shown for species displayed in ordination diagrams.

		1st sampling period		2nd sampling period	
	**Abbreviation**	**Non-crop habitat island**	**Arable land**	**Non-crop habitat island**	**Arable land**
Agonum muelleri (Herbst, 1784)	Agomue	1	6	0	0
Agonum sexpunctatum (Linnaeus, 1758)	Agosex	1	12	1	2
Amara aenea (De Geer, 1774)	Amaaen	14	28	15	7
Amara communis (Panzer, 1797)	Amacom	9	4	25	0
Amara convexior Stephens, 1828	Amacon	19	2	27	0
Amara curta Dejean, 1828		1	1	0	0
Amara familiaris (Duftschmid, 1812)		5	4	1	0
Amara littorea C. G. Thomson, 1857		0	2	0	0
Amara lunicollis Schioedte, 1837		6	1	8	1
Amara makolskii Roubal, 1923		0	0	1	0
Amara montivaga Sturm, 1825		2	13	4	0
Amara municipalis (Duftschmid, 1812)		2	0	0	0
Amara nitida Sturm, 1825		2	1	0	0
Amara ovata (Fabricius, 1792)	Amaova	9	15	23	30
Amara plebeja (Gyllenhal, 1810)		2	1	1	1
Amara similata (Gyllenhal, 1810)	Amasim	12	108	11	67
Amara tibialis (Paykull, 1798)		0	2	0	0
Anchomenus dorsalis (Pontoppidan, 1763)	Ancdor	3	87	4	104
Anisodactylus binotatus (Fabricius, 1787)		0	7	0	0
Badister bullatus (Schrank, 1798)	Badbul	21	1	12	1
Badister lacertosus Sturm, 1815		6	0	4	0
Bembidion lampros (Herbst, 1784)	Bemlam	12	16	0	6
Bembidion obtusum Audinet-Serville, 1821		0	5	0	1
Bembidion properans (Stephens, 1828)		0	2	0	0

	Abbreviation	1st sampling period Non-crop habitat island	1st sampling period Arable land	2nd sampling period Non-crop habitat island	2nd sampling period Arable land
Bembidion quadrimaculatum (Linnaeus, 1761)		1	1	0	0
Brachinus explodens Duftschmid, 1812		1	13	27	0
Calathus fuscipes (Goeze, 1777)	Calfus	47	2	338	16
Calathus melanocephalus (Linnaeus, 1758)	Calmel	42	1	27	2
Carabus cancellatus Illiger, 1798		1	0	0	0
Carabus convexus Fabricius, 1775		7	0	5	0
Carabus granulatus Linnaeus, 1758	Cargra	5	19	3	1
Carabus hortensis Linnaeus, 1758	Carhor	36	0	7	0
Carabus nemoralis O. F. Müller, 1764		16	1	3	0
Clivina fossor (Linnaeus, 1758)		0	1	0	0
Cychrus caraboides (Linnaeus, 1758)		0	0	1	0
Harpalus affinis (Schrank, 1781)	Haraff	2	19	1	2
Harpalus distinguendus (Duftschmid, 1812)		0	2	0	0
Harpalus quadripunctatus Dejean, 1829		10	0	3	0
Harpalus honestus (Duftschmid, 1812)		6	6	3	0
Harpalus latus (Linnaeus, 1758)		19	0	7	0
Harpalus signaticornis (Duftschmid, 1812)		1	5	0	2
Harpalus tardus (Panzer, 1796)	Hartar	43	6	22	0
Laemostenus terricola (Herbst, 1784)		0	1	1	0
Leistus ferrugineus (Linnaeus, 1758)		2	0	2	0
Licinus depressus (Paykull, 1790)		1	0	1	0
Loricera pilicornis (Fabricius, 1775)		1	4	0	1
Microlestes minutulus (Goeze, 1777)		4	0	3	0
Nebria brevicollis (Fabricius, 1792)		0	2	3	1
Notiophilus biguttatus (Fabricius, 1779)		1	0	2	0
Notiophilus palustris (Duftschmid, 1812)		2	0	3	0
Oxypselaphus obscurus (Herbst, 1784)		0	1	0	0
Panagaeus bipustulatus (Fabricius, 1775)	Panbip	9	0	57	0
Philorhizus crucifer (Lucas, 1846)		4	0	0	0
Platynus assimilis (Paykull, 1790)		11	10	5	7

	Abbreviation	1st sampling period Non-crop habitat island	1st sampling period Arable land	2nd sampling period Non-crop habitat island	2nd sampling period Arable land
Poecilus cupreus (Linnaeus, 1758)	Poecup	70	891	25	186
Poecilus versicolor (Sturm, 1824)	Poever	580	186	643	71
Pseudoophonus rufipes (De Geer, 1774)	Pseruf	9	1	19	10
Pterostichus melanarius (Illiger, 1798)	Ptemel	37	91	1829	182
Pterostichus niger (Schaller, 1783)		1	0	17	1
Pterostichus nigrita (Paykull, 1790)		0	8	1	1
Pterostichus oblongopunctatus (Fabricius, 1787)	Pteobl	43	3	40	1
Pterostichus strenuus (Panzer, 1796)	Ptestr	23	11	8	6
Pterostichus vernalis (Panzer, 1796)		3	0	3	2
Syntomus truncatellus (Linnaeus, 1761)		4	0	12	0
Synuchus vivalis (Illiger, 1798)		0	0	5	0
Trechus quadristriatus (Schrank, 1781)		3	7	7	8
