# Supplementary material for: Even the Smallest Non-Crop Habitat Islands Could Be Beneficial: Distribution of Carabid Beetles and Spiders in Agricultural Landscape
Source: PLoS One. 2015 Apr 10;10(4):e0123052. doi: 10.1371/journal.pone.0123052 (PMC4393288; doi:10.1371/journal.pone.0123052)
Supplement: S2 Table — Species highlighted in green were sampled solely within arable land sites (= arable land specialists), species highlighted in red were sampled solely within non-crop habitat islands (= non-crop habitat specialists) and species written in black were sampled in both habitat types (= generalist species). Abbreviations are shown for species displayed in ordination diagrams. (PDF) [file pone.0123052.s003.pdf]

S2 Table

Complete list of recorded spider species classified by their habitat preferences. Species highlighted in green were sampled solely within arable land sites (= arable land specialists), species highlighted in red were sampled solely within non-crop habitat islands (= non-crop habitat specialists) and species written in black were sampled in both habitat types (= generalist species). Abbreviations are shown for species displayed in ordination diagrams.

		1st sampling period		2nd sampling period	
	**Abbreviation**	**Non-crop habitat island**	**Arable land**	**Non-crop habitat island**	**Arable land**
Abacoproeces saltuum		0	0	2	0
Agelena labyrinthica		0	0	1	0
Agroeca brunnea		1	0	1	0
Alopecosa cuneata	Alocun	110	3	8	0
Alopecosa pulverulenta		1	0	1	0
Alopecosa trabalis		13	2	0	0
Araeoncus humilis	Arahum	0	1	0	1
Araniella cucurbitina		1	0	1	0
Aulonia albimana		0	0	2	0
Bathyphantes gracilis		0	0	0	1
Bathyphantes parvulus		0	0	1	0
Centromerus incilium		2	0	0	0
Centromerus sylvaticus		7	0	0	0
Ceratinella brevis		8	0	1	0
Clubiona terrestris		1	0	0	0
Dictyna arundinacea		0	0	1	0
Dicymbium nigrum		1	0	0	0
Diplocephalus picinus		2	0	0	0
Diplostyla concolor	Dipcon	12	0	16	2
Enoplognatha thoracica		12	0	4	1
Erigone atra		0	0	2	1
Erigone dentipalpis		0	1	0	0
Euophrys frontalis		3	0	1	0
Haplodrassus signifer	Hapsig	19	2	12	1

	Abbreviation	1st sampling period Non-crop habitat island	Arable land	2nd sampling period Non-crop habitat island	Arable land
Haplodrassus silvaticus	Hapsil	15	0	24	0
Haplodrassus soerenseni	Hapsoe	59	0	9	0
Haplodrassus umbratilis	Hapumb	50	0	15	0
Harpactea hombergi		1	0	0	0
Harpactea rubicunda	Harrub	168	0	44	0
Heliophanus cupreus		4	0	3	0
Lepthyphantes flavipes		5	0	2	0
Lepthyphantes pallidus		1	0	0	0
Lepthyphantes tenuis		4	0	6	1
Linyphia hortensis		3	0	0	0
Linyphia marginata		0	0	1	0
Macrargus carpenteri		1	0	0	0
Meioneta rurestris		0	0	1	0
Micaria fulgens		5	0	1	0
Micaria pulicaria		9	0	1	0
Microneta viaria		4	0	0	0
Neriene clathrata		4	0	2	0
Oedothorax agrestis		0	0	0	1
Oedothorax apicatus	Oedapi	0	15	6	99
Oedothorax retusus		0	0	0	1
Ostearius melanopygius		2	0	8	1
Ozyptila atomaria		1	0	1	0
Ozyptila claveata		2	0	0	0
Ozyptila praticola	Ozypra	29	0	21	0
Ozyptila trux		1	0	4	1
Pachygnatha degeeri	Pacdeg	26	13	9	16
Pardosa agrestis	Paragr	0	1	1	3
Pardosa lugubris	Parlug	1262	4	269	8
Pardosa palustris	Parpal	12	4	6	4
Pardosa prativaga	Parpra	10	5	2	4

	Abbreviation	1st sampling period Non-crop habitat island	Arable land	2nd sampling period Non-crop habitat island	Arable land
Pardosa pullata		30	0	6	2
Pelecopsis radicicola		1	0	0	0
Phlegra fasciata		0	0	1	0
Phrurolithus festivus		5	0	9	0
Pirata hygrophilus		0	0	1	0
Pocadicnemis juncea		1	0	1	0
Pocadicnemis pumila		1	0	0	0
Porrhomma microphthalmum		0	2	1	0
Robertus arundineti	Robaru	0	11	0	5
Robertus lividus		0	0	1	0
Segestria senoculata		1	0	0	0
Stemonyphantes lineatus		2	0	0	0
Tapinocyboides pygmaeus		5	0	0	0
Tibellus oblongus		1	0	2	0
Tiso vagans		3	0	1	1
Trochosa ruricola	Trorur	0	5	0	0
Trochosa spinipalpis		2	0	0	0
Trochosa terricola	Troter	30	4	21	0
Walckenaeria antica		1	0	0	0
Walckenaeria atrotibialis		0	0	2	0
Walckenaeria vigilax		0	1	0	1
Xerolycosa nemoralis		2	0	1	0
Xysticus audax		1	0	0	0
Xysticus cristatus		3	0	3	0
Xysticus erraticus		7	0	1	0
Xysticus kochi	Xyskoc	4	3	0	5
Zelotes aurantiacus		3	0	1	0
Zelotes clivicola	Zelcli	14	0	6	0
Zelotes electus		1	0	0	0
Zelotes latreillei		5	0	3	1

		1st sampling period		2nd sampling period	
	Abbreviation	Non-crop habitat island	Arable land	Non-crop habitat island	Arable land
Zelotes lutetianus	Zellut	0	4	0	4
Zelotes petrensis		1	0	4	0
Zelotes praeficus	Zelpra	7	0	22	0
Zelotes pusillus	Zelpus	134	7	9	2
Zelotes subterraneus	Zelsub	56	0	37	0
Zora spinimana		2	0	9	0
