# Supplementary material for: Even the Smallest Non-Crop Habitat Islands Could Be Beneficial: Distribution of Carabid Beetles and Spiders in Agricultural Landscape
Source: PLoS One. 2015 Apr 10;10(4):e0123052. doi: 10.1371/journal.pone.0123052 (PMC4393288; doi:10.1371/journal.pone.0123052)
Supplement: S1 Fig — Variance partitioning was based on the following groups of variables: 1st group = sampling period; 2nd group = local environmental conditions (i.e., tree cover, shrub cover, grass cover, litter depth); 3rd group = non-crop habitat island area. A particular group of variables is omitted when it includes no significant variable. Net effects and shared variation (percentage of total variance) explained by particular groups of variables are shown. (DOCX) [file pone.0123052.s004.docx]

**S1 Fig.**

**Relative importance of particular variables affecting the total activity density of carabid beetles within non-crop habitat islands.** Variance partitioning was based on the following groups of variables: 1^st^ group = sampling period; 2^nd^ group = local environmental conditions (i.e., tree cover, shrub cover, grass cover, litter depth); 3^rd^ group = non-crop habitat island area. A particular group of variables is omitted when it includes no significant variable. Net effects and shared variation (percentage of total variance) explained by particular groups of variables are shown.
