# Supplementary material for: Even the Smallest Non-Crop Habitat Islands Could Be Beneficial: Distribution of Carabid Beetles and Spiders in Agricultural Landscape
Source: PLoS One. 2015 Apr 10;10(4):e0123052. doi: 10.1371/journal.pone.0123052 (PMC4393288; doi:10.1371/journal.pone.0123052)

**S7 Fig.**

**The relationship between the activity density of non-crop habitat specialist spider adults and non-crop habitat island grass cover.** Full circles (solid line) represent the first sampling period (spring to early summer) and open circles represent the second sampling period (peak summer).


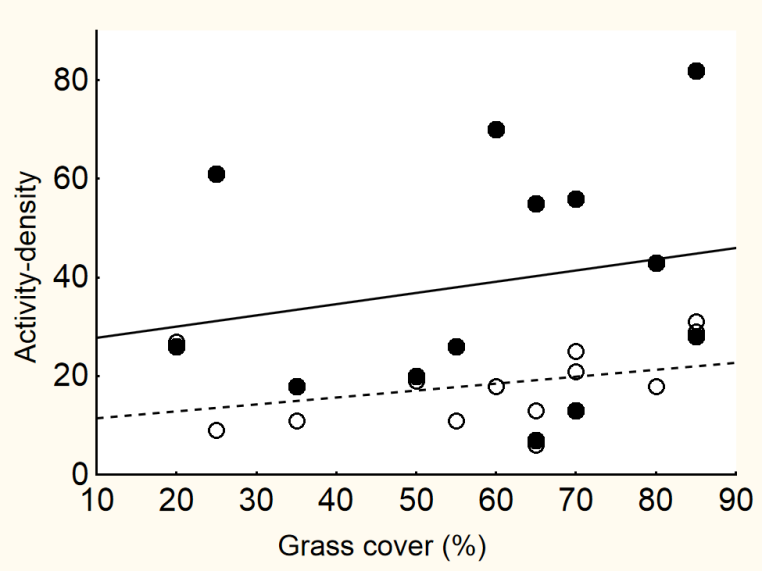

Supplement: S7 Fig — Full circles (solid line) represent the first sampling period (spring to early summer) and open circles represent the second sampling period (peak summer). (DOCX) [file pone.0123052.s010.docx]
