# Supplementary material for: Even the Smallest Non-Crop Habitat Islands Could Be Beneficial: Distribution of Carabid Beetles and Spiders in Agricultural Landscape
Source: PLoS One. 2015 Apr 10;10(4):e0123052. doi: 10.1371/journal.pone.0123052 (PMC4393288; doi:10.1371/journal.pone.0123052)

**S10 Fig.**

**The effects of sampling period, shrub cover, grass cover, tree cover and habitat island area on the species composition of non-crop habitat specialist spider assemblages recorded within non-crop habitat islands.** The ten most influential species for the analysis (CCA) are displayed and named using abbreviations made from the first three letters of the genus name followed by the first three letters of the species name (for full names see S2 Table).


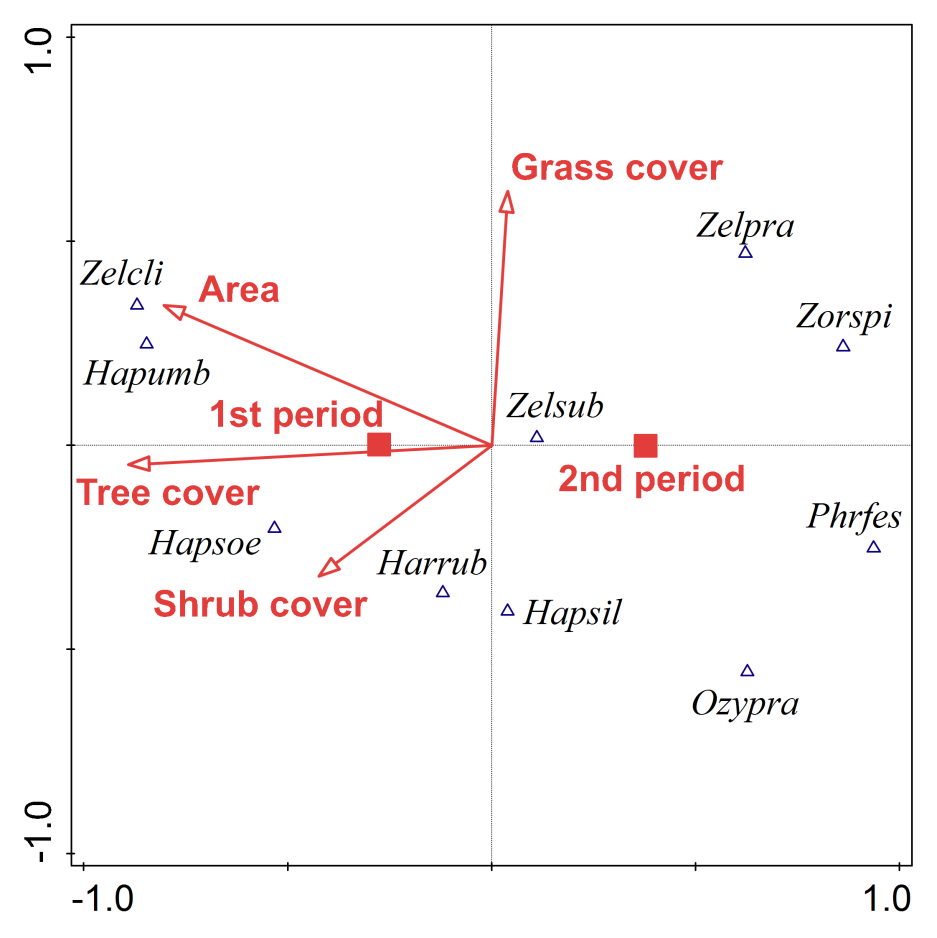

Supplement: S10 Fig — The ten most influential species for the analysis (CCA) are displayed and named using abbreviations made from the first three letters of the genus name followed by the first three letters of the species name (for full names see S2 Table). (DOCX) [file pone.0123052.s013.docx]
