# Supplementary material for: Even the Smallest Non-Crop Habitat Islands Could Be Beneficial: Distribution of Carabid Beetles and Spiders in Agricultural Landscape
Source: PLoS One. 2015 Apr 10;10(4):e0123052. doi: 10.1371/journal.pone.0123052 (PMC4393288; doi:10.1371/journal.pone.0123052)
Supplement: S11 Fig — The relationships between tree cover (treecov), grass cover (grasscov), shrub cover (shrubcov), litter depth (litter) and non-crop habitat island area (logarea) are displayed. The matrix was generated using “pairs.panels” function from the “psych” package for R. (DOCX) [file pone.0123052.s014.docx]

**S11 Fig.**

**The correlation matrix of non-crop habitat island characteristics investigated in this study.** The relationships between tree cover (treecov), grass cover (grasscov), shrub cover (shrubcov), litter depth (litter) and non-crop habitat island area (logarea) are displayed. The matrix was generated using “pairs.panels” function from the “psych” package for R.
